# Up- and Downregulated Genes after Long-Term Muscle Atrophy Induced by Denervation in Mice Detected Using RNA-Seq

**DOI:** 10.3390/life13051111

**Published:** 2023-04-29

**Authors:** Shoko Sawano, Misaki Fukushima, Taiki Akasaka, Mako Nakamura, Ryuichi Tatsumi, Yoshihide Ikeuchi, Wataru Mizunoya

**Affiliations:** 1 Department of Animal and Marine Bioresource Sciences, Graduate School of Agriculture, Kyushu University, Fukuoka 819-0395, Japan; 2 Department of Food and Life Science, School of Life and Environmental Science, Azabu University, Sagamihara 252-5201, Japan; 3 Department of Animal Science and Biotechnology, School of Veterinary Medicine, Azabu University, Sagamihara 252-5201, Japan

**Keywords:** skeletal muscle, atrophy, denervation, gene expression, RNA-Seq, fiber type, sarcopenia

## Abstract

Skeletal muscle atrophy occurs rapidly as a result of inactivity. Although there are many reports on changes in gene expression during the early phase of muscle atrophy, the patterns of up-and downregulated gene expression after long-term and equilibrated muscle atrophy are poorly understood. In this study, we comprehensively examined the changes in gene expression in long-term denervated mouse muscles using RNA-Seq. The murine right sciatic nerve was denervated, and the mice were housed for five weeks. The cross-sectional areas of the hind limb muscles were measured using an X-ray CT system 35 days after denervation. After 28 d of denervation, the cross-sectional area of the muscle decreased to approximately 65% of that of the intact left muscle and reached a plateau. Gene expression in the soleus and extensor digitorum longus (EDL) muscles on the 36th day was analyzed using RNA-Seq and validated using RT-qPCR. RNA-Seq analysis revealed that three genes—*Adora1, E230016M11Rik*, and *Gm10718*—were upregulated and one gene—*Gm20515*—was downregulated in the soleus muscle; additionally, four genes—*Adora1*, *E230016M11Rik*, *Pigh*, and *Gm15557*—were upregulated and one gene—*Fzd7*—was downregulated in the EDL muscle (FDR < 0.05). Among these genes, *E230016M11Rik*, one of the long non-coding RNAs, was significantly upregulated in both the muscles. These findings indicate that *E230016M11Rik* could be a candidate gene for the maintenance of atrophied skeletal muscle size and an atrophic state.

## 1. Introduction

Skeletal muscles are specialized organs that produce dynamic physical forces and undergo structural and functional changes in response to mechanical loading and contractile activity. Skeletal muscle atrophy is caused by factors such as inactivity [1], aging [2], food deprivation [3], disease [4], and denervation [3,5,6,7,8,9]. However, the molecular mechanisms underlying its regulation are not completely understood. An improved understanding of the molecular regulation of muscle atrophy would help in improving general health by preventing muscle wastage and related injuries. To that end, numerous studies have investigated comprehensive gene expression changes at the early phase in atrophic muscles induced by denervation [7,9], fasting, diabetes mellitus, renal failure, tumor implantation [10], and hind-limb suspension [11]. These studies revealed the genes related to protein catabolic processes that were commonly upregulated.

A comparison of gene expression between atrophic and control muscles led to the identification of a subset of genes, typically up- or downregulated, that are thought to regulate the loss of muscle mass. These atrophy-related genes are referred to as atrogenes [12]. Many previous studies have shown that muscle atrophy is an active process regulated by specific signaling pathways and transcriptional programs. Two of the most prominent identified genes involved in muscle atrophy were *atrogin-1* and *MuRF1* [13,14]. These two muscle-specific ubiquitin ligases are commonly upregulated in various models of muscle atrophy and are involved in the increased degradation of myofibrillar proteins by the ubiquitin-proteasome system.

Sacheck et al. performed microarray experiments in atrophied muscles following sciatic nerve denervation and spinal cord isolation in rats and found that the expression of *atrogin-1*, a muscle-specific ubiquitin ligase, increased and decreased to control levels after 14 days [7]. By contrast, muscle weight decreased after 14 days, which suggested that atrophy-related genes other than muscle-specific ubiquitin ligases are involved. However, it is not fully understood which genes are induced or repressed in atrophying muscles. Among atrophy-related genes, *FoxO1* and *metallothionein 1* (*Mt1*) are known to exhibit persistent upregulation after 14 days [7]. FoxO1 is a forkhead-type transcription factor and activates the expression of atrophy-related genes, such as *atrogin-1* and *cathepsin L*, in various muscle atrophy-related conditions [15,16]. Mt1 is a protein, belonging to the metallothionein family of proteins, that regulates the homeostasis of essential trace metals such as zinc and copper [17]. *Mt1* is upregulated in multiple types of skeletal muscle atrophy [10,18] and is suggested to protect muscle cells from oxidative stress [19], although the effects on muscle atrophy are unknown. In this study, we used *FoxO1* and *Mt1* as marker genes of muscle atrophy.

Numerous recent transcriptomic studies have identified a large number of RNAs classified as long non-coding RNAs (lncRNAs). The number of human lncRNA genes published by meta-assembly, referred to as the FANTOM CAGE-associated transcriptome (FANTOM CAT) [20] and MiTranscriptome compendium [21], which are independent research projects, is approximately 27,000 and 59,000, respectively, which exceeds the number of protein-coding genes. Among all the genes registered in GENCODE, the percentage of genes for which some literature information exists is more than 70% for protein-coding genes but less than 2% for lncRNA genes. Thus, 98% of lncRNA genes have no literature information, which implies that the functions of most lncRNAs have not been clarified [22].

There are two major fiber types in skeletal muscles—slow- and fast-twitch fibers [23]. Slow-twitch fibers are rich in mitochondria, possess high oxidative capacity, and are resistant to fatigue. Muscles enriched in slow-twitch fibers perform sustained and tonic contractile activities such as postural tension. Conversely, fast-twitch fibers exhibit high glycolytic metabolism and fatigue. The soleus and extensor digitorum longus (EDL) muscles are hind limb muscles. In several mammals, the soleus is predominantly composed of slow-twitch fibers, whereas the EDL predominantly comprises fast-twitch fibers. Thus, these two muscles (slow and fast muscles) are often used as representative slow- and fast-twitch fibers, respectively [24]. It is known that the effects of muscle atrophy are not the same in fast- and slow-twitch fibers [6,25,26]. Accordingly, we compared the soleus and EDL muscles in this study.

Here, we examined whole mRNA expression using RNA-Seq to determine the genes that show fluctuating expression levels in sufficiently atrophied soleus and EDL muscle tissues of mice. We found that atrophy of both muscles equilibrated 36 days after denervation. In this state, several up- or downregulated genes were newly detected in each muscle, and the commonly regulated genes were assumed to be related to a new set point that maintained skeletal muscle size in the atrophic state.

In this study, we found two lncRNA candidate genes relating to long-term muscle atrophy. Our findings suggest a new concept: that lncRNAs may have a role in maintaining atrophied skeletal muscle size and condition.

## 2. Materials and Methods

### 2.1. Animals

Eight male C57BL/6J mice (10-weeks old), purchased from KBT Oriental, Tosu, Japan, were housed at 22 ± 2 °C and 55 ± 10% humidity on a 12:12 light/dark cycle and received water and the AIN-93G diet ad libitum during the 1-week equilibration period. All animal experiments were conducted in strict accordance with the recommendations of the Guidelines for Proper Conduct of Animal Experiments published by the Science Council of Japan. Ethics approval was obtained from the Kyushu University Institutional Review Board (approval no. A26-078 and A28-090).

### 2.2. Sciatic Nerve Transection

The mice were intraperitoneally anesthetized with a combination of anesthetics—0.3 mg/kg medetomidine, 4.0 mg/kg midazolam, and 5.0 mg/kg butorphanol. The right hind limb was prepared for surgery, a skin incision was made along the femoral axis, and a 1-mm sciatic nerve transection was performed unilaterally in anesthetized mice. The sciatic nerve in the left hind limb was not amputated; however, a sham operation was performed and used as the control.

### 2.3. Measurement of Muscle Cross-Sectional Area Using an X-ray CT System

**Figure 1:**
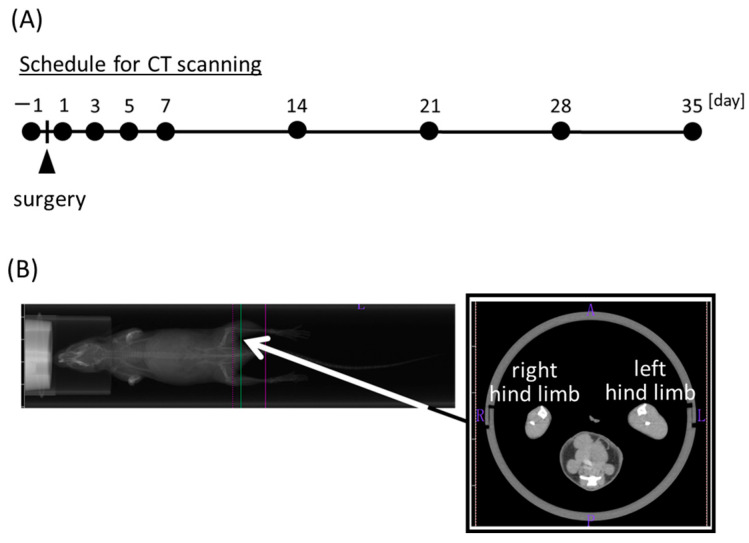
Measurements of cross-sectional area of mouse hind limbs via X-ray CT scanning for 35 days. (**A**) Time schedule for X-ray CT analysis, including pre- and post-denervation. The images were taken at daily intervals until one week postoperatively and then every week thereafter. (**B**) Typical CT scan imaging. The total areas of the gray color in left and right hind limbs were measured as skeletal muscle areas.

The muscle cross-sectional area was measured using an X-ray CT system (Latheta LCT-100). Anesthesia inhalation equipment (NARCOBIT for Isoflurane) was used to keep the mice anesthetized, supine, and anteriorly immobilized. The maximum cross-sectional area was recorded on days −1, 1, 3, 5, 7, 14, 21, 28, and 35 after denervation to estimate muscle mass (Figure 1A). Measurements were taken at a width of 0.5 mm, and approximately 30 slices were obtained from the area between the knee and ankle (Figure 1B). The photographed lower hind limb muscle cross-sections were used to calculate the maximum cross-sectional area of the skeletal muscle using Latheta 3.00A software.

### 2.4. RNA Preparation and Next-Generation Sequencing (RNA-Seq)

Soleus and EDL muscles were obtained immediately after the mice were euthanized by decapitation following isoflurane inhalation anesthesia on day 36 after denervation. Total RNA was isolated from the excised soleus and EDL muscles using the RNeasy Fibrous Tissue Mini Kit (QIAGEN, Hilden, Germany), and RNA Integrity Number was checked. The extracted total RNA (n = 8) was pooled to equilibrate its amount. The pooled total RNA was submitted to Otogenetics Corporation (Atlanta, GA, USA) for RNA-Seq assay. Briefly, the integrity and purity of total RNA were assessed using an Agilent Bioanalyzer and the OD_260/280_ values. The cDNA (500 ng) was generated from 100 ng total RNA using the Clontech SmartPCR cDNA kit (Clontech Laboratories, Inc., Mountain View, CA, USA). The cDNA was fragmented using Covaris (Covaris, Inc., Woburn, MA, USA), profiled using an Agilent Bioanalyzer and subjected to Illumina library preparation using the NEBNext reagent (New England Biolabs, Ipswich, MA, USA). The quality, quantity, and size distribution of Illumina libraries were determined using Agilent 2100 Bioanalyzer. The libraries were then subjected to Illumina HiSeq2500 sequencing according to standard protocols. Paired-end 101–126 nucleotide reads were generated to reach depths of 58,303,244, 38,051,509, 41,128,489, and 39,771,306 reads for control soleus, denervated soleus, control EDL, and denervated EDL, respectively.

### 2.5. Sequence Mapping and Counting

Quality control of the sequencing reads was performed using FastQC [27], and quality trimming was performed using PRINSEQ [28]. In this step, raw reads were filtered by removing reads with Phred base quality scores below 20 and reads shorter than 40 bases. Simultaneously, reads were processed by trimming sequences from the 3′-end with the threshold scores of Phred base quality scores below 30 and 15 bases at the 5′-end to remove positional sequence bias. All downstream analyses were based on the trimmed reads. In the read mapping step, the reads were aligned with the reference genome (GRCm38 (GCA_000001635.4)). Read mapping was performed using TopHat2, which employs the widely used Bowtie2 program as the alignment engine [29,30]. The mapped reads were counted using HTSeq [31]. Data were analyzed using DESeq for differential expression [32]. Genes with more than ten reads were subjected to differential expression analysis for reliable quantification. Here, we chose a cost-effective sample pooling strategy to use with the RNA-Seq. DESeq allows the analysis of experimental results with no biological replicates. While one may not draw strong conclusions from this analysis, it can still be useful for exploration and hypothesis generation. Next, we validated the differential expression and examined biological variations using quantitative reverse transcriptase-PCR (RT-qPCR).

### 2.6. Gene Ontology (GO) Term Enrichment Analysis

DAVID 6.8 online tool was used to identify the significant GO terms and clusters [33,34]. The list of ENSEMBL gene ID was uploaded into DAVID. Enrichment values (GO Terms), enrichment scores (annotation clusters), and statistical determinants (Fisher’s exact *p*-values and Benjamini) were calculated using DAVID. A Fisher’s exact *p*-value = 0 represents perfect enrichment. Usually, the *p*-value must be ≤0.05 to be considered strongly enriched in the annotation categories. Benjamini is an adjusted *p*-value defined as the smallest significance level for which the given hypothesis would be rejected, when the entire family of tests is considered.

### 2.7. RT-qPCR

RT-qPCR was performed using the Roche LightCycler 1.5 PCR system and TaqMan probe detection format. First, cDNA was synthesized from total RNA using SuperScript III (18080–044; Invitrogen, Grand Island, NY, USA) and oligo (dT) primers (H09876; Roche, Mannheim, Germany). The primer sets for the genes analyzed are listed in Table 1 and were designed using the Roche Universal Probe Library Assay Design Center (ProbeFinder version 2.35 for mouse) with an intron-spanning assay. The mRNA expression levels of mouse *FoxO1*, *Mt1*, *Adora1*, *E230016M11Rik_1*, *Pigh*, *Frizzled-7 (Fzd7)*, *Gm15557*, *Gm20515*, and *Gm10718* were measured. Threshold cycle (Ct) was defined as the lowest number of PCR cycles after which an increase in fluorescence above the baseline signal could be detected. The annealing temperature was set to 60 °C in all cases. Genes were analyzed using a standard curve constructed with serial dilutions of cDNA aliquots pooled from one randomly chosen sample. Gene expression was calculated relative to that of *Gapdh* and expressed as fold change.

### 2.8. Statistical Analysis

Data are presented as mean ± SE. Statistically significant differences from the control group at *p* < 0.05 and *p* < 0.01 are indicated on graphs by (*) and (**), respectively. Comparisons were performed using Student’s *t*-test for two experimental groups. Statistics were calculated using Excel Toukei 2006 (Social Survey Research Information Co., Ltd., Tokyo, Japan). For differential gene expression analysis using RNA-Seq, statistical significance was determined using the false discovery rate (FDR). FDR < 0.05 was considered as significant and indicated on graphs by (**).

## 3. Results

### 3.1. Chronological Measurement of Muscle Cross-Sectional Area Using X-ray CT System

**Figure 2:**
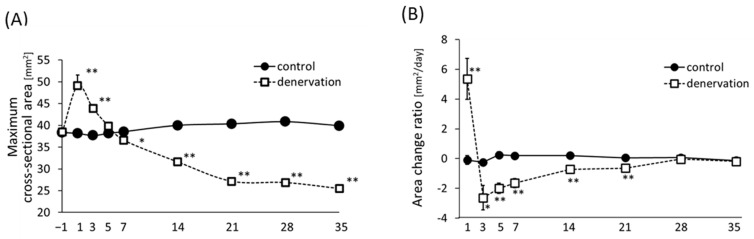
Time-course analysis of muscle cross-sectional area and area change ratio of mouse hind limb measured using an X-ray CT system. (**A**) Muscle cross-sectional area at days −1, 1, 3, 5, 7, 14, 21, 28, and 35 after denervation treatment is shown. Values are calculated from total cross-sectional areas of an X-ray CT system. (**B**) The graph shows the extent of daily change in cross-sectional areas, which were calculated from alterations between measurements. *: *p* < 0.05, **: *p* < 0.01 compared to the same time control group using unpaired Student’s *t*-test. Error bars: SE, n = 8.

To trace the alterations in muscle size after denervation, the cross-sectional area of the hind limb muscle was chronologically measured for 36 days. We used an X-ray CT scanner to measure the maximum cross-sectional area of the leg muscles. Results showed that the cross-sectional area of the lower hind limb muscles temporarily increased on day 1 after denervation compared to that of the innervated control muscles (Figure 2A). Thereafter, the muscle cross-sectional area of the denervated muscles decreased over time, and by day 7, the cross-sectional area was less than that of the control muscles; on day 21, the muscle cross-sectional area decreased to approximately 65% of that of the control muscles. Subsequently, it plateaued and exhibited almost no further change. Figure 2B shows the change in the muscle cross-sectional area per day. The largest decrease was observed after 3 days of denervation. After day 3, the range of variation decreased gradually; after day 28, as in the control muscles, no change in the muscle cross-sectional area was observed (Figure 2B).

**Figure 3:**
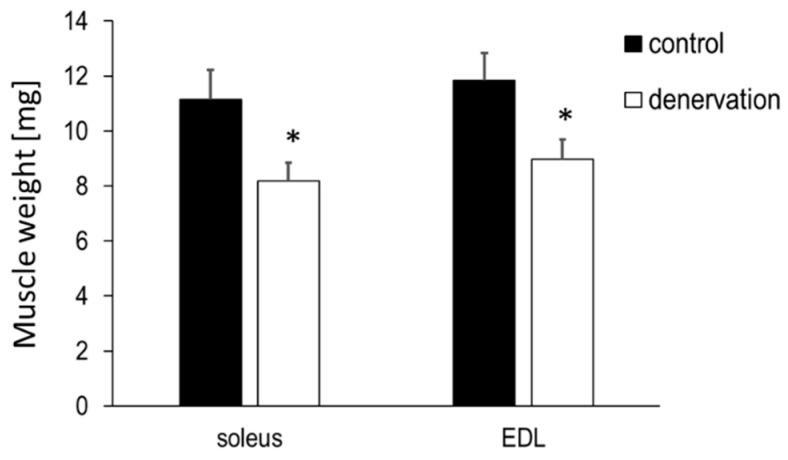
Skeletal muscle weight of soleus and EDL after long-term denervation. The black columns show the muscle weight of the control leg without denervation, and the white columns show the muscle weight of the denervated leg 36 days after denervation. Values are expressed as mean ± SE. * *p* < 0.05, compared to the control group using unpaired Student’s *t*-test. n = 8.

On day 36 after denervation, the skeletal muscles were removed, and muscle weight was found to be significantly reduced compared to that of the control sham-operated innervated muscles, with the weight of soleus muscles decreasing to 73% and that of the extensor digitorum longus (EDL) muscles decreasing to 76% of the weight of control muscles (Figure 3).

### 3.2. Expression of Genes Related to Atrophy in Skeletal Muscles

**Figure 4:**
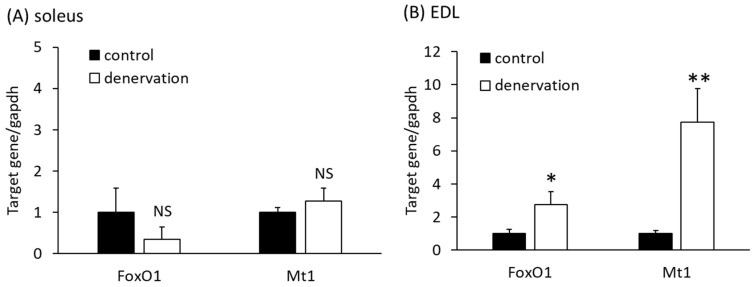
mRNA levels of muscle atrophy-related genes (*FoxO1* and *Metallothionein 1* (*Mt1*)) in soleus (**A**) and EDL (**B**) muscles 36 days after denervation measured using RT-qPCR. *Gapdh* was used as the endogenous control gene. Each bar represents mean ± SE (n = 8). * *p* < 0.05, ** *p* < 0.01 compared with the control group using Student’s *t*-test.

We first examined the expression levels of *FoxO1* and *Mt1*, which are known to be related to atrophy, prior to a comprehensive study of skeletal muscle gene expression using RNA-Seq. After 5 weeks of denervation, on day 36, when muscle atrophy was considered to have reached a plateau, the soleus and EDL muscles were resected, and the expression levels of *FoxO1* and *Mt1* were analyzed. Each muscle showed differential gene expression. In slow soleus muscles, *FoxO1* and *Mt1* expression did not differ from that in the controls (Figure 4A). On the other hand, in the EDL muscle (fast muscle), both *FoxO1* and *Mt1* were significantly upregulated after denervation (Figure 4B).

### 3.3. RNA-Seq Analysis of Muscles with Long-Term Atrophy Induced by Denervation

**Figure 5:**
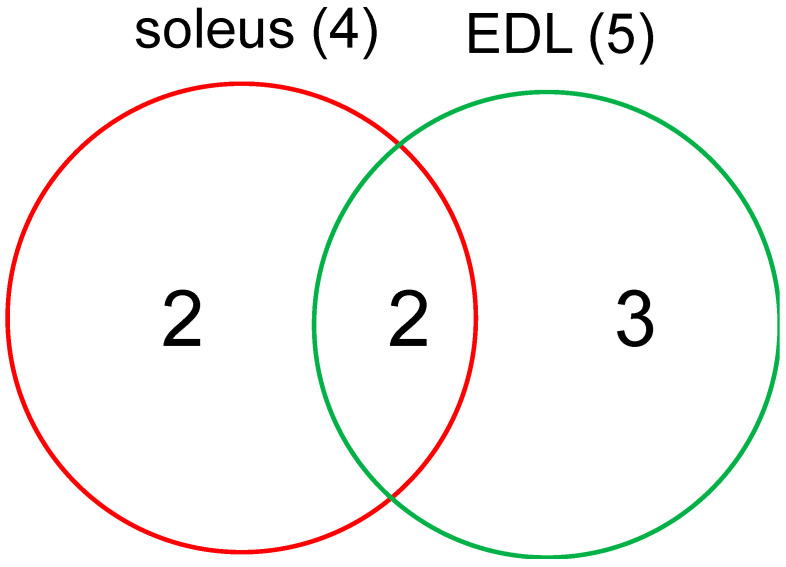
Venn diagram showing the number of unique and common differentially expressed genes between soleus and EDL muscles.

**Figure 6:**
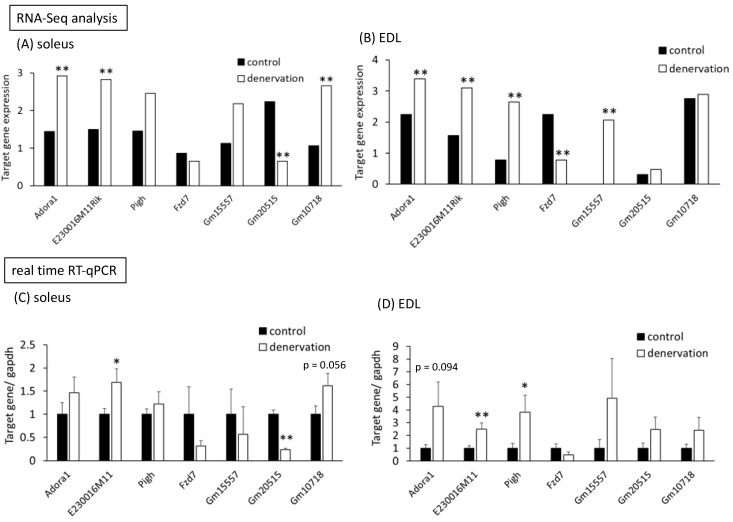
Expression levels of selected genes determined using RNA-Seq and RT-qPCR analysis. (**A**,**B**) Columns show the target gene expression in soleus (**A**) and EDL (**B**) muscles. Each value represents the normalized read count by DESeq and presented as log_10_. **: *p* < 0.01 and FDR < 0.05, n = 1 (pooled 8 samples/group). (**C**,**D**) Validation of RNA-Seq data using RT-qPCR in soleus (**C**) and EDL (**D**) muscles. *Gapdh* was used as the endogenous control gene. Each bar represents mean ± SE (n = 8). * *p* < 0.05, ** *p* < 0.01 compared with the control group by Student’s *t*-test.

An RNA-Seq assay was performed using the total RNA derived from the soleus and EDL muscles 36 days after sciatic nerve transection. The summary of sequencing results is given in Table S1. Most reads were mapped on the genome. The raw read counts of genes processed using HTSeq in each treatment group are shown in Table S2. After mapping and analyzing the RNA-Seq dataset, gene expression in the denervated muscles was compared to that in the control muscles (sham-operated). Of the total 37,315 genes, those with significant variations in expression are listed in Table 2. In the soleus muscle, four genes were significantly regulated (FDR < 0.05) in the denervated muscles; these were *Adora1*, *E230016M11Rik*, *Gm20515*, and *Gm10718*. Of these, *Adora1*, *E230016M11Rik*, and *Gm10718* were upregulated, whereas *Gm10718* was downregulated by long-term soleus muscle atrophy (Figure 5 and Figure 6A). By contrast, five genes—*Adora1*, *E230016M11Rik*, *Pigh*, *Fzd7*, and *Gm15557*—were significantly differentially expressed in the denervated EDL muscles; *Fzd7* was downregulated, and *Adora1*, *E230016M11Rik*, *Pigh*, and *Gm15557* were upregulated (Figure 5 and Figure 6B).

Of these genes, *Adora1* and *E230016M11Rik* were significantly upregulated by long-term denervation in both soleus and EDL muscles, whereas the other genes were independently regulated in each muscle (Table 2 and Figure 5 and Figure 6A,B).

We found that a set of seven significantly up- and downregulated genes was enriched in molecules known or predicted to be involved in integral component of membrane, transmembrane helix, transmembrane, TRANSMEM:Helical, and membrane (Table 3). There were no enrichments in KEGG pathways.

### 3.4. RT-qPCR Analysis of Genes in Muscles with Long-Term Atrophy Induced by Denervation

To confirm the validity of RNA-Seq results, we performed RT-qPCR analysis of the seven aforementioned genes in soleus and EDL muscles, i.e., *Adora1*, *E230016M11Rik*, *Pigh*, *Fzd7*, *Gm15557*, *Gm20515*, and *Gm10718*. In the soleus muscles, *E230016M11Rik* was significantly upregulated and *Gm20515* was significantly downregulated, similar to the RNA-Seq results (Figure 6C). *Gm10718* tended to be upregulated by denervation compared to that in the control group (*p* = 0.056, Figure 6C). Furthermore, in the denervated EDL muscles, *E230016M11Rik* and *Pigh* were significantly upregulated compared to those in the control muscles, and *Adora1* tended to be upregulated (*p* = 0.094) (Figure 6D).

## 4. Discussion

In this study, we used RNA-Seq to determine the genes with fluctuating expression levels in skeletal muscles that are stabilized after denervation-induced atrophy in mice. We found that muscle atrophy progressed to a plateau without further muscle atrophy. The up- or downregulated genes were inferred to define a new set point in which skeletal muscle size is maintained in an atrophic state. The effects of skeletal muscle atrophy include not only a decrease in muscle weight but also a decrease in cross-sectional area and muscle volume. Therefore, to investigate the time at which muscle atrophy reaches a plateau state, we measured the cross-sectional area using an X-ray CT system, which allows for continuous and non-invasive observation.

The cross-sectional area of the lower hind limb muscles examined using an X-ray CT system showed a temporary increase in the muscle cross-sectional area immediately after denervation (Figure 2A), which was attributed to edema caused by motor nerve injury. A lack of muscle contractile capacity due to motor nerve injury prevents fluid egression from the interstitial spaces, ultimately leading to edema. Such symptoms develop within weeks of nerve injury or surgical denervation in humans and are considered a hallmark of neuritis, similar to those which occur in brachial neuritis [35]. The edema lasted for up to 5 days after denervation in mice, after which the cross-sectional area clearly decreased. To determine when muscle atrophy ceased, we calculated the daily change in the cross-sectional area; we found that the cross-sectional area decreased over time and that the decrease was greatest after three days of denervation. These changes have been demonstrated in previous studies, which reported a marked increase in muscle weight immediately after denervation treatment, followed by a gradual slowing down [7].

Upon further confirmation with muscle weight measurements of the skeletal muscles, the weight of excised soleus and EDL muscles showed similar reduction, which was 73 and 76% of that in the control intact muscle, respectively. We measured food intake and body weight during the experimental period and found that daily food intake remained unchanged at 3–5 g after denervation, and that body weight showed a gradually increasing trend at approximately 26 g. These results confirmed that the reduction in skeletal muscle weight due to denervation was not a consequence of malnutrition.

To comprehensively examine the gene expression in skeletal muscles removed on day 36, we first investigated the expression levels of *FoxO1* and *Mt1*, which are considered atrogenes, using RT-qPCR. These atrogenes remained differentially expressed 14 days after denervation [7]. We examined whether these genes remained upregulated after 36 days of denervation. No changes in expression were observed in the soleus, whereas *FoxO1* and *Mt1* were significantly upregulated in the EDL. These results suggested that although the soleus (slow-twitch fiber-predominant) and EDL (fast-twitch fiber-predominant) muscles showed similar levels of atrophy, different intracellular mechanisms contributed to atrophy.

The two most notable atrogenes are the muscle-specific ubiquitin ligases *atrogin-1* and *MuRF1*, whose expression is elevated in various states of muscle atrophy and is involved in promoting protein degradation by the ubiquitin-proteasome system [13,14]. Indeed, mice deficient in either *atrogin*-*1* or *MuRF1* are partially resistant to denervation-induced atrophy [13]. To date, these two genes are the best markers of muscle atrophy that have been identified and can be considered the master genes of muscle atrophy. However, our RNA-Seq results showed no increase in the expression of *atrogin-1* or *MuRF1* after 36 days of denervation. Interestingly, according to Sacheck et al., the expression of most atrogenes, such as those involved in denervation and spinal cord isolation, returns to baseline levels 14 days after disuse-induced treatment [7]. This suggests that these atrogenes play an important role in early atrophy processes.

As shown in Table 2, RNA-Seq analysis revealed increased expression of three genes (*Adora1*, *E230016M11Rik*, and *Gm10718*) and decreased expression of one gene (*Gm20515*) in the soleus muscle and increased expression of four genes (*Adora1*, *E230016M11Rik*, *Pigh*, and *Gm15557*) and decreased expression of one gene (*Fzd7*) in the EDL muscles. Two of these genes (*Adora1* and *E230016M11Rik*) were upregulated in both the muscle tissues. The results of RT-qPCR confirmed a similar trend in gene expression as observed in RNA-Seq results. Using RNA-Seq, we detected only seven significantly differentially up- or downregulated genes (FDR < 0.05), from which *FoxO1* and *Mt1* were excluded. The reason for the low number of differentially expressed genes is probably the limited detection sensitivity without biological replicates. However, this also indicates that the genes detected here can be expected to be strongly involved in the atrophy of muscles.

Functional enrichment analysis is employed for gene expression data analysis to detect cellular mechanisms such as molecular function, biological processes, and crucial biological pathways affected by gene expression alterations according to conditions. Functional annotation clustering using DAVID suggested that differentially expressed genes in this experiment are associated with transmembrane proteins. Among differentially expressed genes, *Adora1* and *Fzd7* are genes to code transmembrane proteins. In atrophic muscles, myofibrillar proteins are mainly degraded and proteolytic systems are highly active. However, when the atrophy reaches a plateau, the transmembrane molecules may become more important in maintaining the cellular condition.

Adora1 is an adenosine receptor belonging to the G protein-coupled receptor 1 family (a transmembrane protein). There are four known types of adenosine receptors in humans—Adora1, Adora2A, Adora2B, and Adora3—each with specific ligand-binding patterns and tissue distribution, which regulate diverse physiological functions. Adora1 is highly expressed in the brain (cerebral cortex, cerebellum, and hippocampus) [36,37] and peripheral tissues such as adipose tissue [38]. It has been reported that Adora1 stimulation in adipose tissue increases the expression of the adipogenic marker FABP4 and reduces that of the brown adipose tissue marker UCP1 [39]. Furthermore, in vitro studies have shown that the genetic or acute pharmacological activation of Adora1 induces adipogenesis and reduces lipolysis in adipocytes. Adora1 has been reported to be expressed in skeletal muscles [40,41], but its function in that context remains unknown. Muscular Adora1 may also play a role in regulating cellular metabolism, similar to that in adipose tissue. To date, there have been no reports suggesting that Adora1 is involved in skeletal muscle physiological traits.

*E230016M11Rik* and *Gm20515* are lncRNAs in the mammalian genome [42,43,44]. The lncRNAs are thought to perform diverse physiological functions by binding to various RNA-binding proteins, resulting in modifications of subcellular localization, protein-protein interactions, and regulation of the enzyme activities of these RNA-binding proteins. By contrast, miRNAs exhibit physiological functions by regulating mRNA expression. Thus, elucidating the physiological functions of lncRNAs is more complicated than elucidating those of miRNAs. In this study, *E230016M11Rik* and *Gm20515* were found to be involved in muscle atrophy for the first time; however, the underlying mechanisms are unknown. A recent study showed the important role of lncRNA *Gm10561* in myogenesis [45], which was upregulated during myogenic differentiation and is highly expressed in skeletal muscle. In that study, authors indicated the function of *Gm10561* as a competing endogenous RNA (ceRNA) for miRNA *miR-432*. *miR-432* directly targets *MEF2C* and *E2F3*, which are known regulators of muscle development. Similarly, *E230016M11Rik* and *Gm20515* may regulate long-term muscle atrophy by interacting with certain signal molecules that regulate muscle mass. Further studies are required to confirm this hypothesis.

*Pigh* expression was increased in the denervated EDL muscle 36 days after denervation. Phosphatidylinositol glycan anchor biosynthesis genes are involved in the synthesis of glycosylphosphatidylinositols (GPI) and their transfer to proteins to act as lipid bilayer anchors. GPI is synthesized in the endoplasmic reticulum and is attached to the carboxyl terminus of a protein with a GPI-attachment signal sequence. The function of Pigh in the skeletal muscle is not yet understood, but a study by Furlow et al. [5] listed *Pigh* as one of the genes whose expression significantly increased after 14 days of sciatic nerve denervation in mice. Although its function remains unknown, this protein is thought to play a role in muscle atrophy.

*Fzd7* is a transmembrane receptor for Wnt signaling proteins. Maltzahn et al. observed that *Wnt7a* binding to *Fzd7* directly activates the Akt/mTOR growth pathway in differentiated myofibers, thereby inducing muscle fiber hypertrophy [46]. Thus, the downregulation of *Fzd7* in the EDL muscle may result in a decrease in Wnt signaling and anabolic pathway activities, leading to a reduction in skeletal muscle mass. Moreover, it is known that the Wnt7a/Fzd7 signaling pathway plays an important role in promoting the symmetrical expansion of muscle satellite cells through the planar cell polarity (PCP) pathway [47] and in enhancing the migration of muscle satellite cells [48]. However, to the best of our knowledge, this is the first report showing that *Fzd7* expression decreases during muscle atrophy.

It is known that the effects of muscle atrophy are not the same in fast- and slow-twitch fibers, although there was no marked difference between the soleus and EDL weights 36 days after denervation in our experiment. In general, slow-twitch fibers are more sensitive to inactivity, microgravity, and denervation-induced atrophy, whereas type 2 fibers are more vulnerable to cancer cachexia, diabetes, chronic heart failure, and aging [6,25,26]. The basis of the selectivity of fiber-type specific muscle atrophy remains an important unresolved issue. Gao and Li revealed differences in various cellular signaling pathways, such as Akt activation, between denervated mouse soleus and EDL muscles [49]. Several significantly differently up- or downregulated genes were detected between the soleus and EDL muscles in our study. This difference may be due to different signaling molecules and transcriptional responses in denervation-induced atrophy.

There are mainly two limitations in this study. First, the absence of biological replicates in RNA-Seq. An increase in biological replicates will provide us with more robust conclusions, and more abundant genes which are statistically significantly different are expected to be detected. Second, it is necessary to examine protein expression to validate our mRNA data. Some antibodies against Adora1, Pigh, and Fzd7 are commercially available. Hence, we can use Western blotting or ELISA to examine the expression level of these proteins. These limitations should be considered in future studies.

Taken together, the results of the present study show that muscle atrophy plateaued on day 36 after denervation. Transcriptome analysis showed that the expression of different genes fluctuated in both soleus and EDL denervated muscles, suggesting the involvement of different atrophy mechanisms. *Adora1* and *E230016M11Rik* were upregulated in the long-term atrophic soleus and EDL, which suggests their function in maintaining reduced skeletal muscle size and condition regardless of the muscle fiber type. Further studies on the gain- and loss-of-function mutations of these genes are required.

## Figures and Tables

**Table 1:** PCR primer sets used for quantitative reverse transcriptase-PCR.

Gene	Primers	
***FoxO1***	L: cttcaaggataagggcgaca	R: gacagattgtggcgaattga
***metallothionein 1***	L: caagtgcacctcctgcaa	R: ttcgtcacatcaggcacag
***Adora1_1***	L: tcctcacccagagctccat	R: gagtcaccactgtcttgtaccg
***E230016M11Rik_1***	L: gctcttaaccgctgagcaa	R: ggccagtctgagcatctagaaa
***Pigh***	L: tacgggctcttcaccctgt	R: gaggtaaccaagcaggcctaa
***Fzd7***	L: gccatttgacttgaaacttgg	R: tccgccttctctccttgag
***Gm15557***	L: tcaggaatggggttagagga	R: gtgtataaacagtacgaggacatggt
***Gm20515***	L: tcaataaggggcaccatttc	R: cgccacatgtatgttttgatg
***Gm10718***	L: aattttccacctttttctgtcct	R: tgaaaaatgagaaatgcacactg
***Gapdh***	L: gggttcctataaatacggactgc	R: ccattttgtctacgggacga

**Table 2:** Genes regulated by long-term atrophy in soleus and EDL muscles.

	Genes	Soleus	EDL
*Adora1*	adenosine A1 receptor	↑	↑
*E230016M11Rik*	(non-coding RNA)	↑	↑
*Pigh*	phosphatidylinositol glycan anchor biosynthesis, class H	−	↑
*Fzd7*	frizzled class receptor 7	−	↓
*Gm15557*	(Known protein coding)	−	↑
*Gm20515*	(non-coding RNA)	↓	−
*Gm10718*	(Known protein coding)	↑	−

**Table 3:** Functional annotation clustering using DAVID for the up- and downregulated genes induced by long-term atrophy in soleus and EDL muscles.

Annotation Cluster	Enrichment Score: 1.18	#Genes	*p*-Value	Benjamini
GOTERM_CC_DIRECT	integral component of membrane	4	2.9 × 10^−2^	7.7 × 10^−1^
UP_KW_DOMAIN	Transmembrane helix	4	5.5 × 10^−2^	1.0 × 10^−1^
UP_KW_DOMAIN	Transmembrane	4	6.9 × 10^−2^	1.0 × 10^−1^
UP_SEQ_FEATURE	TRANSMEM:Helical	4	8.3 × 10^−2^	3.4 × 10^−1^
UP_KW_CELLULAR_COMPONENT	Membrane	4	1.3 × 10^−1^	5.4 × 10^−1^

## Data Availability

We have uploaded the raw data of RNA-Seq to DNA Data Bank of Japan (DDBJ) website (https://www.ddbj.nig.ac.jp/index-e.html, accessed on 13 March 2023).

## Supplementary Materials

The following supporting information can be downloaded at: https://www.mdpi.com/article/10.3390/life13051111/s1, Table S1: Summary of sequencing results of control/denervated soleus and EDL muscles from mice; Table S2: The raw read count of genes processed by HTSeq in each treatment group.
