# Supplementary material for: Up- and Downregulated Genes after Long-Term Muscle Atrophy Induced by Denervation in Mice Detected Using RNA-Seq
Source: Life (Basel). 2023 Apr 29;13(5):1111. doi: 10.3390/life13051111 (PMC10221910; doi:10.3390/life13051111)
Supplement: Supplementary file 1 [file life-13-01111-s001.zip › Table S1.pdf]

Table S1. Summary of sequencing results of control/denervated soleus and EDL muscles from mice.

Treatment		Raw reads	Timmed reads	Total mapped reads	Total mapping %	Q30% a	GC content% b	Sequence length
Con-Sol	left	58,303,244	58,292,880	58,289,696	99.98	97.23	45	101
	right	58,303,244	58,237,008	58,225,759	99.97	96.49	44	101
Den-Sol	left	38,051,509	38,042,590	38,039,842	99.97	97.29	46	106
	right	38,051,509	37,981,112	37,959,449	99.76	96.22	46	106
Con-EDL	left	41,128,489	41,119,913	41,116,883	99.97	98.16	46	126
	right	41,128,489	41,100,986	41,031,763	99.76	97.27	46	126
Den-EDL	left	39,771,306	39,763,013	39,760,332	99.97	98.12	46	126
	right	39,771,306	39,750,885	39,683,722	99.78	97.46	46	126

a: Percentage of reads with Phred values greater than 30, b: Percentage of guanine and cytosine base numbers of total bases.
